# Recent findings in the genetics and epigenetics of asthma and allergy

**DOI:** 10.1007/s00281-019-00777-w

**Published:** 2020-02-14

**Authors:** Michael Kabesch, Jörg Tost

**Affiliations:** 1 Department of Pediatric Pneumology and Allergy, St. Hedwig’s Hospital of the order of St. John, University Children’s Hospital Regensburg (KUNO), Steinmetzstr. 1-3, 93049 Regensburg, Germany; 2 Laboratory for Epigenetics and Environment, Centre National de Recherche en Génomique Humaine, CEA - Institut de Biologie François Jacob, 2 rue Gaston Crémieux, 91000 Evry, France

**Keywords:** Asthma, Allergy, Genetics, Epigenetics, Interaction

## Abstract

In asthma and allergy genetics, a trend towards a few main topics developed over the last 2 years. First, a number of studies have been published recently which focus on overlapping and/or very specific phenotypes: within the allergy spectrum but also reaching beyond, looking for common genetic traits shared between different diseases or disease entities. Secondly, an urgently needed focus has been put on asthma and allergy genetics in populations genetically different from European ancestry. This acknowledges that the majority of new asthma patients today are not white and asthma is a truly worldwide disease. In epigenetics, recent years have seen several large-scale epigenome-wide association studies (EWAS) being published and a further focus was on the interaction between the environment and epigenetic signatures. And finally, the major trends in current asthma and allergy genetics and epigenetics comes from the field of pharmacogenetics, where it is necessary to understand the susceptibility for and mechanisms of current asthma and allergy therapies while at the same time, we need to have scientific answers to the recent availability of novel drugs that hold the promise for a more individualized therapy.

## Introduction

Asthma and allergy genetics were dominated by genome-wide association studies (GWAS) for more than a decade. Starting with the first GWAS on asthma by our GABRIEL consortium in 2007 [1], numerous publications followed, exploring genetic susceptibility for elevated total IgE [2], allergic sensitization [3], atopic dermatitis [4], and allergic rhinitis [5] as well as food allergy [6]. Over the years, the consortia investigating these phenotypes grew bigger and bigger, allowing to find marginal associations of odds ratios lower than 1.2, but still with strong *p* values, due to the sheer force of numbers. The last of these studies included well over 100,000 cases [7].

This era has now come to an end. Common genetic traits for common diseases have been largely identified. However, missing heritability in asthma and allergy is still high, and even ever-larger numbers of patients in GWAS studies will not increase knowledge on genetic susceptibility as the technique as used today has reached its limit of resolution.

On the other hand, the analysis of epigenetic modifications in allergic diseases has recently attracted substantial interest, as epigenetic modifications might mediate the effects of the environment on the development of or protection from allergic diseases as well as constitute a novel class of biomarkers and potentially provide new therapeutic targets [8, 9]. Epigenetics, which includes DNA methylation, posttranslational histone modifications, nucleosome occupancy, and small and long noncoding RNAs, may indeed hold the key to explaining the high degree of plasticity of the immune response throughout life.

Rather than focusing on ever larger studies of ill-defined phenotypes (such as asthma per se and general allergic sensitization), the field is currently moving into new directions and towards new system-medicine technologies with artificial intelligence looming on the horizon to make use of massive multi-layer data derived from genomic, epigenomic, transcriptomic, and metabolomics approaches that are collected now. In this review, we focus on current trends in genetics and epigenetics of allergic diseases.

### Current trends in asthma and allergy genetics and epigenetics

In genetics, a trend towards three main topics in asthma and allergy genetics developed over the last 2 years. Studies on overlapping and/or very specific phenotypes within the allergy spectrum but also reaching beyond, looking for common genetic traits shared between different diseases or disease entities. Furthermore, asthma and allergy genetics in populations genetically different from European ancestry have now been performed. This is extremely necessary, as the majority of new asthma patients today are not white and asthma is a worldwide disease with more than 230 million people affected across all races and continents according to WHO. In epigenetics, several large-scale epigenome-wide association studies (EWAS) have been published and recent studies focus on the interaction between the external and internal (e.g., the microbiome) environment and epigenetic signatures extending our knowledge to novel environmental factors and mechanism of disease.

Finally, the major trend in current asthma and allergy that unites genetics and epigenetics, comes from the field of pharmacogenetics, driven by the recent availability of novel drugs that hold the promise for a more individualized therapy. However, these biologicals come at a prize that makes it financially necessary for the health system of almost any country to better understand the mechanisms of disease and to better manage the distribution of these new drugs specifically to those in greatest need and likely to benefit.

### The genetic susceptibility for more specific asthma and allergy phenotypes

**Fig. 1:**
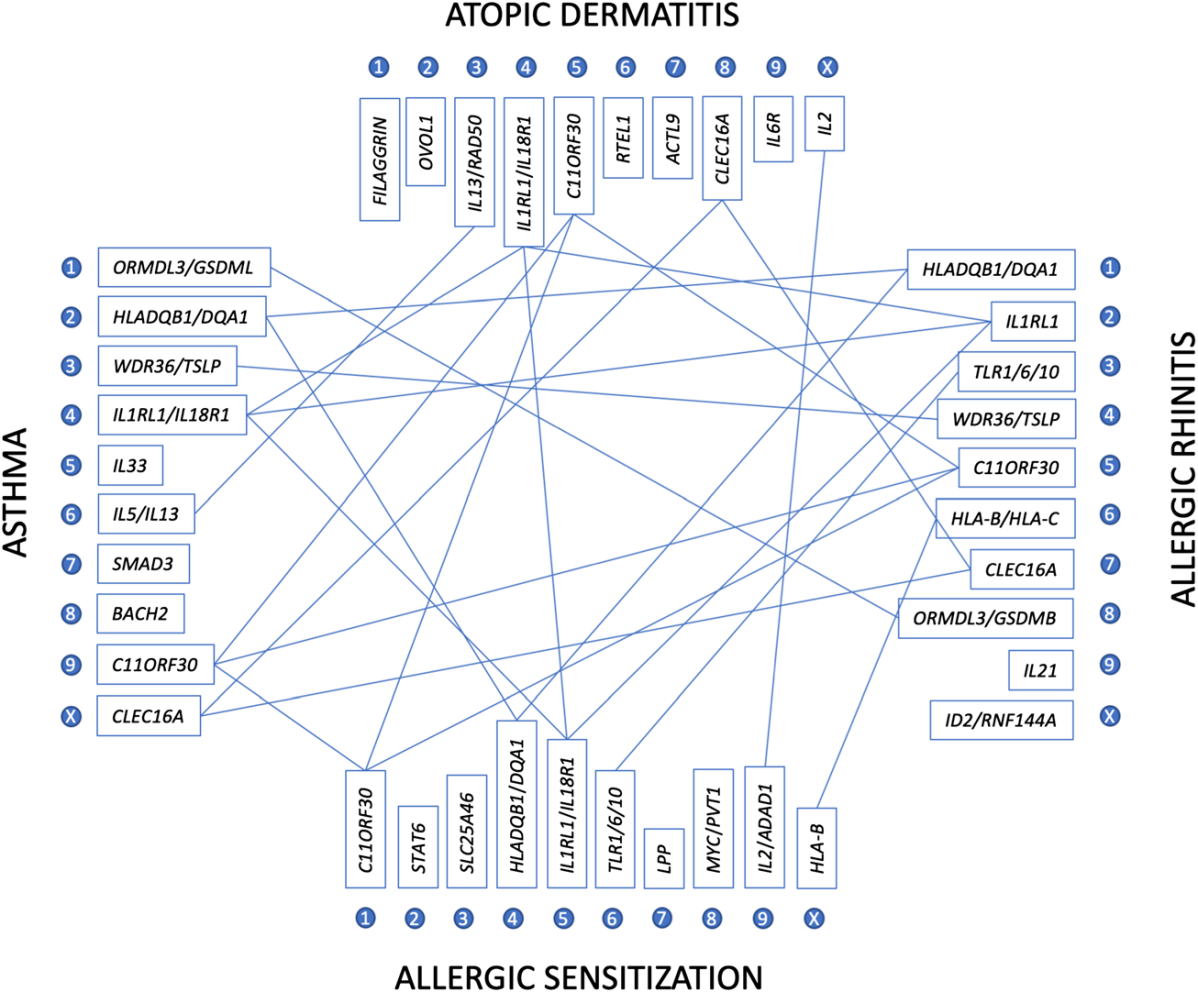
Genes involved in different allergic diseases. Top ten genes associated with the respective allergic disease in most recent and extensive GWAS as described in the text were compared for overlaps in their associations. The more links a gene has, the more general the role of its mutations in allergic mechanisms may be

About 100 years ago, it was first noticed that atopic diseases such as asthma, allergic rhinitis, and atopic dermatitis occur overproportionally frequent in some families and even in the same patient. It came quite as a surprise, when the first GWAS were published on asthma [1], total IgE [2], atopic dermatitis [4], allergic sensitization [3], and allergic rhinitis [5], that many hits and genes for these diseases were not shared. It took some time and much larger datasets to identify the indeed existing overlaps between allergic diseases (Fig. 1). Finally, in 2018, on the basis of the UK biobank and an enormous effort in genotyping and bioanalysis, about 30 shared genetic loci were identified across the genome [10]. When expression analyses were performed on respective hit genes, a vast majority of these genes were found to be expressed in the skin but not so much in other tissues, suggesting that the skin could be the primal battleground for the development of the different allergic diseases. It could be hypothesized that genetic alterations of the skin barrier may facilitate an unnatural presentation of allergens to the immune system and thus, starting allergic reactions, later expressed in different organs such as the skin, the airways, and the gut (or combinations thereof).

A further recent study not focusing on pleiotrophy but gene environment interaction is also worth mentioning in this context. Traffic-related air pollution was found to be associated with atopic dermatitis in children in the presence of a genetic risk background [11], which was previously already associated with the development of asthma in connection with air pollution from environmental tobacco smoke and traffic related air pollution [12]. Calculating weighted genetic risk scores from a total of nine polymorphisms in four candidate genes (*GSTP1*, *TNF*, *TLR2*, and *TLR4*) were associated. These findings were based on 6 birth cohort studies from Europe and Canada and suggest that interaction between genetic susceptibility for inflammation and increased reaction to pollution on the one hand, and early life exposure to traffic on the other, can increase the risk for atopic dermatitis in children, while such an association between air pollution and atopic dermatitis was not observed for those without genetic susceptibility. Although not investigated in this study, such an association could also be expected for asthma, taken the data from Zhu [10] into account. Thus, new and exciting evidence from genetics points towards the skin to have a gatekeeper function for the development of allergic diseases in general on the basis of environmental exposures and genetic susceptibility.

### Genetic pleiotrophy for comorbidities with asthma and allergy

**Fig. 2:**
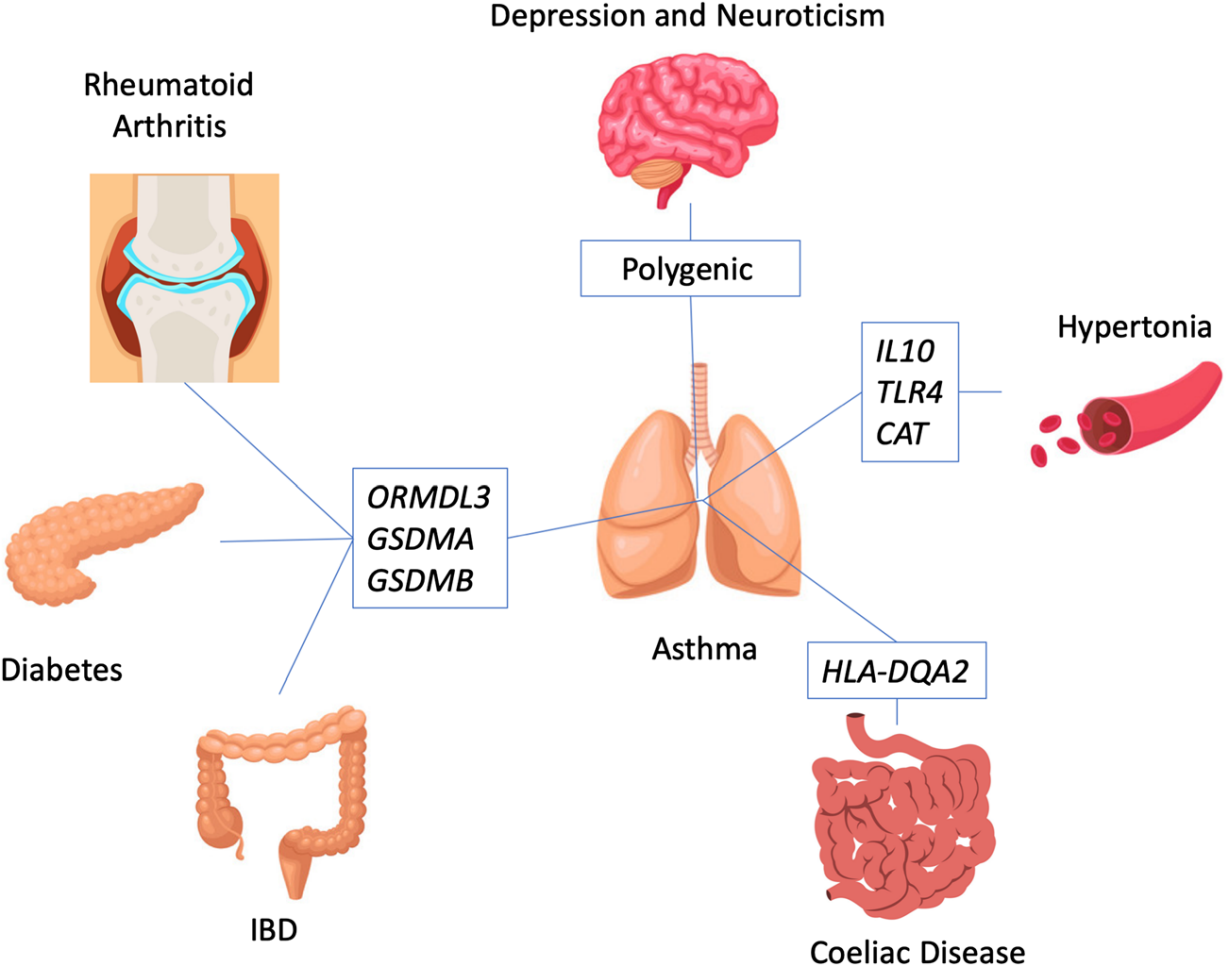
Genes linking different diseases to asthma. Candidate genes identified to be associated with asthma and allergy have also been implicated in other diseases. These genes and the associated diseases are depicted

Pleiotrophy was also identified between asthma and a number of other diseases (Fig. 2). The most consistent finding in asthma genetics is the association between asthma starting in childhood and a risk locus on chromosome 17q21. Interestingly, that same region was furthermore associated with ulcerative colitis [13] and Crohn’s disease [14] and susceptibility to type I diabetes and rheumatoid arthritis were reported while we did not find an association with multiple sclerosis [15]. In large twin populations from Scandinavia, genetic traits for asthma overlapped with those from different affective disorders such as major depression disorder, primary anxiety disorder, and most of all, neuroticism [16]. Using sophisticated bioinformatics tools, also an overlap between gene networks contributing to asthma and hypertension based on genetic databases and bioinformatic ranking algorithms was identified [17]. The genes most likely in the center of the asthma and hypertension interaction were *IL10*, *TLR4*, and *CAT*, suggesting that mechanisms of adaptive and innate immunity are shared in the development of both diseases. In addition, association between asthma and celiac disease was observed, but only when a genetic background for asthma in the family was present in children with an atopic form of asthma [18]. The overlapping association clustered to the HLA system, known to play a role in celiac disease for a long time and linked to asthma in a recent massive scale GWAS [7].

### Asthma and allergy genetics in non-Caucasian populations

First GWAS on asthma and allergic diseases were all performed in Caucasian populations, and therefore, it was not clear if these results could easily be transferred to populations of other genetic backgrounds or may even be of any use in these populations. First GWA studies in African American populations followed and the results suggested that indeed, a somewhat different genetic architecture for allergic diseases may be present [19]. In mostly small and underpowered studies only two loci associated with asthma in African American were known until recently: *PTGS* on chromosome 9q34 [20] and *PYHIN1* on chromosome 1 [21]. This was also due to the fact that genotyping chips specific for African genetic ancestry and imputation tools for that ancestry were not available. Only in 2019, the first large-scale GWAS on asthma in African Americans was published by the Consortium on Asthma among populations of African Ancestry in the Americas (CAAPA) [22]. In a landmark effort, the consortium first sequenced almost 900 individuals of African ancestry to create an imputation base. Then, the novel ADPC (African Diaspora Power Chip), to complement previous GWAS chips for specific African SNPs was developed. Finally, pooling smaller previous study populations, an analysis based on approximately 7.500 cases and as much controls was created. The results indicate that some of the genetic background for asthma is shared between Caucasians and African Americans when 11 out of 18 major asthma associations were confirmed. In addition, three African specific association signals were identified and these three belong to the top 5 asthma signals in the study. The most significant association signal comes however from the well-known 17q21 locus.

In this study, it also became evident that there is also a need to better understand the genetic influence of Native American populations on the development of asthma and allergy. The reason for that is the massive increase of children with asthma and allergic diseases in Central and South America as shown by recent ISAAC surveys [23]. As CAAPA drew their African American probands from numerous populations across the Americas with different degrees of admixture, the authors did find hints of Native American influences in their analyses, but they could only speculate about the true role of that background in the development of asthma and allergies. CAAPA is a blueprint for further GWAS studies urgently needed also in Asian, South American, and African populations. This may come as a surprise especially when thinking about all the Chinese and Japanese studies that have already been performed in the field, also recently [24, 25]. These studies are state of the art and of considerable size. For example, the recent meta-analysis of 29 case-control studies suggested a somewhat more important role of FceRIß polymorphisms in the development of asthma and allergic rhinitis in Asian populations compared to Caucasians [25]. Performed with tools centered around European backgrounds, they are only capable to confirm if signals identified in Europeans can also be found in Asians, but they cannot find specific factors contributed by Asian backgrounds. What is needed now is a profound and serious approach to the topic as demonstrated so impressively by CAAPA.

### EWAS for asthma and allergy

In contrast to the genetic field, several general EWAS have only been published recently (Table 1). Most of these were still using the old 450K chip but some (e.g., [33]) were already done with the current EPIC BeadChip (Illumina, Inc.) interrogating either 450,000 or 840,000 of the 29 M CpGs in the human genome. In the EWAS from the MEDALL consortium using four European birth cohorts and validating 14 CpGs in further seven cohorts, childhood asthma was found to be associated with a number of differentially methylated CpG positions in whole blood [26]. In particular, the analysis of a subgroup of individuals for whom purified circulating eosinophils were available showed an altered DNA methylation profile suggesting a differential activation state and that changes observed in blood are probably largely driven by this cell population. The importance of this cell type was further shown in the ALSPAC cohort, where none of the initially ~ 300 significant CpGs remained significant after adjustment for eosinophil and neutrophil cell count estimates [34]. The so-far largest cross-sectional EWAS (631 cases and 2231 controls) using nine different cohorts increased the number of differentially methylated CpGs associated with asthma to 179 CpGs and 36 regions [27]. In general, there is significant overlap of the findings in EWAS analyzing asthma or atopy [31]. EWAS have shown to explain better the variation in a phenotype than GWAS as demonstrated for, e.g., circulating IgE levels [35] and levels of asthma related proteins such as *CHI3L1* are partly mediated by DNA methylation changes, but not genetic variation [36].

**Table 1:** Main characteristics of recent large-scale epigenome-wide association studies. All listed studies used the Infinium HumanMethylation450 BeadArray (485,000 CpGs covering all genes). Diff: differentially; DMR: differentially methylated region; EVA-PR: Epigenetic Variation and Childhood Asthma in Puerto Ricans; PIAMA: Prevention and Incidence of Asthma and Mite Allergy birth cohort; SLSJ: Saguenay-Lac-Saint-Jean asthma familial collection (Quebec)

Study	Allergy	Sample size	Tissue and study design	Main findings	Replication	Comments
Xu et al. [26]	Asthma (2/3 criteria: Doctor diagnosis, recent asthma medication, recent wheezing)	207 cases vs 610 controls at age 4–5; 185 patients vs 546 controls at age 8 (4 European birth cohorts)	Whole blood/cross-sectional	27 CpGs including *LMAN2*, *STX3*, *LPIN1*, *DICER1*	247 cases and 2949 controls (seven cohorts) 14/14 CpGs replicated	DNA methylation changes correlated with eosinophil numbers, more pronounced DNA methylation changes in isolated eosinophils
Reese et al. [27]	Asthma (doctor diagnosis and current or recent asthma episode or recent asthma medication)	631 cases vs 2231 controls 7–17 years (9 cohorts)	PBMCs/cross-sectional	179 CpGs and 36 regions including *ZFPM1* and *IL5RA*	Meta-analysis, changes replicated in purified eosinophils (SLSJ cohort)	Largest meta-analysis so far, includes cohorts of mixed and African American ancestry
		668 cases vs 2904 controls at birth	Cord blood/prospective	9 CpGs associated with: *CLNS1A*, *miR548*, *GPATCH2*, *LOC100129858*, *AK091866*, *SUB1*, *WDR20* and 35 regions	–	DNA methylation changes at birth might predict future asthma risk
Forno et al. [28]	Atopy (positive IgE to 1/5 aeroallergens)	312 cases vs. 171 controls (age 9–20, EVA-PR)	Nasal brushes/cross-sectional	8664 CpGs, top CpGs associated with epithelial barrier function	72 African American children [29], 432 Dutch children (PIAMA), subset with CD326 positive epithelial cells 28/30 of top CpGs replicated,	Replication across different ethnicities 1570 genes with expression changes and diff. methylation
	Atopic asthma (doctor diagnosis and recent wheezing)	169 cases vs. 104 controls (age 9–20, EVA-PR)		28/30 of top CpGs replicated		
Cardenas et al. [30]	Asthma (doctor diagnosis and recent asthma episode or medication)	65 cases vs. 463 controls at age 13	Nasal swabs/cross-sectional	Asthma 285 CpGs Allergic asthma 1235 CpGs FeNO 8372 CpGs Medication 130 CpGs	Data comparison with two published cohorts (Inner city cohort [29], EVA-PR cohort [31]) 50–60% of CpGs replicated	CpGs and DMRs annotated to Th2 activation and eosinophils and some previously associated with Asthma or IgE
Nicodemus-Johnson et al. [32]	Asthma (doctor diagnosis and recent asthma medication)	74 cases vs. 41 controls (average age 40 years)	Cultured endobronchial epithelial cells	40,893 CpGs including CpGs in *ORMDL3*	–	Enrichment of mQTLs in genes with diff. methylation or asthma related genetic risk factors

Most EWAS so far performed have a cross-sectional design; they do thus not allow to distinguish if the observed changes are preceding the onset of the disease (and are probably disease causing) or a consequence of the disease. The PACE consortium analyzed in addition to the above described cross-sectional study also newborn blood DNA methylation in 668 cases and 2904 controls and identified 9 CpGs and 35 differentially methylated regions associated with asthma later on life [27]. While these CpGs represent a potential biomarker for the prediction of asthma later in life, these CpGs have not been associated with asthma in other (cross-sectional) cohorts, which makes it currently difficult to assess their value. Nonetheless, candidate gene studies of Th2 lineage genes and EWAS of limited size already showed the potential of analyses in cord blood predicting asthma at a later age [37, 38]. Notably, methylation changes in the distal promoter of *SMAD3,* an important regulator in T cell differentiation, were replicated in three small cohorts [37].

In general, there is significant overlap of the findings in EWAS analyzing asthma or atopy [31]. Overall, despite the huge advances in the last years, there is still considerable heterogeneity in the published studies. A recent review on EWAS studies in asthma identified among the thousands of CpGs associated with asthma in recent years, that only 41 of the associations were identified in at least one other study [31]. The epigenetic landscape is specific for a given cell thus requiring careful selection of the cell type of relevance for a given biomedical question as well as taking potential confounding effects caused by differential cell composition between, e.g., patients and controls into account [39]. Most studies, especially most of the large EWAS cohort studies (Table 1) have been performed in whole blood or PBMCs, with a few using also sorted blood cell populations albeit in cohorts of limited size. However, an increasing number of studies have also been performed in nasal epithelial cells [28–30]—as a proxy for airway epithelial cells, which are especially in children difficult to collect—and more recently in airway smooth muscle cells [40]. Studies in the nasal epithelial cells identified hundreds to thousands of significant CpGs, showing thus much stronger effects as the EWAS performed in the blood. Of note, most of the top CpGs identified in nasal epithelial cells replicated well in other cohorts analyzing this tissue type despite different ethnicity of the children of the different cohorts [28, 30]. Differentially methylated CpGs included mainly hypomethylated genes regulating eosinophilic and Th2 responses [11]. Further support for the importance of selecting the right cell type comes from an EWAS in atopic dermatitis, where statistically significant DNA methylation changes were only found in skin samples, but not in blood or sorted blood cell populations [41]. In general, DNA methylation changes in tissues other than blood did correlate better with gene expression changes [29, 40] and magnitude of changes and effect sizes of genes that were also found in blood-based EWAS were increased in nasal cells compared to whole blood, but not to sorted eosinophils [30]. Similarly, in the African American inner-city children cohort, changes in the PBMCs were small in magnitude (median 1.3%, range 0.02–3.1%), while those in nasal epithelial cells ranged from 2.6 to 29.5% with a median of 9.5% [29, 42]. Furthermore, there is a substantial overlap of DNA methylation changes observed in nasal cells with DNA methylation changes observed in cultured endobronchial epithelial cells from asthmatics and controls [32]. Furthermore, some genes previously found in blood-based EWAS were confirmed in the nasal cells including *ACOT7*, *EPX*, *GJA4*, and *METTL1*. A predictive model based on DNA methylation changes of 30 CpGs in nasal cells showed improved performance compared to the Asthma Predictive Index to predict development of asthma in children with wheeze [28]. It is likely that significant findings in blood based EWAS reflect the contribution of eosinophils to the disease, while nasal cells represent methylation changes in the airway cells constituting thus two different angles of view on asthma.

The epigenome is determined by the genome and genetic variation and epigenetic variation influence each other [43]. A large proportion of the CpGs in the human genome are implicated in Methylation Quantitative Trait Loci (mQTLs), i.e., the methylation level is at least partly determined by genetic variants in *cis* or in *trans*. However, the proportion of the variance in the methylation levels explained by genetic variation is in most cases rather limited [44]. Studies analyzing epigenetic and genetic variation at large scale in the same individuals are so far limited in allergic diseases. mQTLs were found enriched in cultured endobronchial epithelial cells from a large cohort of asthmatics and controls in genes showing genetic variation and differential DNA methylation associated with asthma [32]. Similarly, 500 CpG-SNP interactions in *cis* were found to be associated with allergic rhinitis and 274 CpG-SNPs with allergic rhinitis associated with asthma [45]. In a recent study, interactions between CpGs and SNPs in the vicinity of about 2/3 of all human genes were investigated and 12 genes associated with asthma with significant CpG-SNP interactions were identified, including three previously described asthma genes (*PF4*, *ATF3*, *TPRA1*) [46].

### Environment and epigenetics in asthma and allergy

Epigenetics might mediate the effects on the environment on cellular homeostasis and contribute to the development of asthma and allergic diseases. DNA methylation changes have been associated with atopy and serum IgE levels [35, 47], and shown to differ between allergic patients with asthma [8, 26, 27, 30], atopic dermatitis [41], food allergy [33, 48, 49], and seasonal allergic rhinitis [50] when compared to healthy individuals. For seasonal allergic rhinitis, DNA methylation alterations show increased discriminatory power compared to gene expression-based signatures and this during as well as outside the allergy season [50]. In the same line, it was recently shown that baseline DNA methylation levels in a gene called *SLFN12* predicted the severity of the allergic reaction when allergic rhinitis patients were exposed to grass pollen [51]. Similarly, analysis of histone modifications or microRNA expression has been shown to detect differences at both the candidate gene level or in genome-wide analyses in allergic individuals [52, 53].

Air pollution, ozone, cigarette smoking, viral infections, use of antibiotics and antipyretics, pets, a traditional farm environment and exposure to mold or dust mites, consumption of raw or unprocessed cow’s milk have been associated with increased or reduced frequency of allergic diseases and have recently been reviewed in detail [8]. We will therefore focus only on recent results. It is well known that prenatal smoking in mothers lead to widespread changes in DNA methylation patterns [54]. A recent study now addressed how these changes compare to changes induces by active smoking and found that the changes in utero caused by prenatal smoking in mothers are more pronounced than those caused by passive smoking after birth or active smoking in teenagers [55]. However, although cigarette smoking has a well proven impact on the development of respiratory allergies and leads to clear and reproducible changes in the DNA methylome, it is of note that hardly any of the smoking associated changes have also been associated with asthma in different EWAS performed.

Air pollution has been linked to lung pathologies such as asthma and has been shown in multiple studies to have a direct impact on genome-wide DNA methylation patterns leading mostly to a loss of DNA methylation [56]. Air pollution and especially particulate matter 2.5 (PM_2.5_) has been shown to alter DNA methylation patterns already in utero [57] and in a recent study using a mouse model for allergic rhinitis PM_2.5_ exposure led to more pronounced symptoms and increased DNA methylation at the *IFNγ* promoter in CD4^+^ T cells suggesting an increased shift towards the Th2 subtype [58]. Exposure to diesel exhaust particles led to much more pronounced changes in the DNA methylation profile when combined with exposure to an allergen within 4 weeks compared to either allergen or particle exposure alone or even simultaneous exposure to both insults suggesting that timing between the insults is of great importance for functional consequences [59]. Air pollution has notably been shown to have a direct influence on the expression of enzymes involved in the balance of DNA methylation and demethylation through increasing DNA methylation levels at the promoter of the DNA demethylating enzyme *TET1* [60]. Recent data in a mouse model deficient for *Tet1* supports an important role for this enzyme in airway disease leading to increased expression of Th1 and Th2 cytokines, lung eosinophilia and airway hyperresponsiveness, which were at least partially mediated by a genome-wide hypermethylation including genes involved in interferon signaling [61]. Furthermore, air pollution has also shown to alter the expression profile of miRNAs involved in inflammation and which have also been associated with allergic diseases [52, 62].

Vaccination has been associated with increased circulating IgE levels and therefore postulated to lead to an increased risk of asthma and other allergies [63]. There is, however, little support for this hypothesis in the literature [64]. In a recent EWAS using the Isle of Wright cohort [65], methylation of two CpGs near immune related genes was associated with tetanus vaccination at genome-wide significance and the two of them were also associated with a decreased risk of asthma. This data is also supported by previous experiments performed in experimental models of food allergy, where treatment of mice with *Heliobacter pylori* lysate or its immunomodulatory peptide VacA led to attenuated anaphylaxis upon challenge probably though a mechanism involving reduced DNA methylation in the Treg-specific demethylated region in the *Foxp3* gene leading to a larger number activated T regulatory cells (Tregs) [66].

One of the most prominent external factors influencing DNA methylation changes is aging and the chronological age can be deduced from the DNA methylation patterns [67]. Accelerated epigenetic aging, i.e., a higher biological age predicted from the DNA methylation patterns then the true chronological age, has been associated with a large number of disease and an overall greater risk of death [67], while longevity has been associated with decelerated epigenetic aging [68]. Epigenetic aging has now also been assessed in the context of atopic or allergen sensitization and asthma using a variety of different clocks [30, 69]. Accelerated epigenetic aging in children at 7–8 years of age was associated with increased serum IgE levels and a 1.2–1.3-fold increased risk of atopic sensitization, or sensitization to environmental or food allergens for every 1-year increase in epigenetic age [69].

### Pharmacogenetics and pharmacoepigenetics of asthma and allergy

**Fig. 3:**
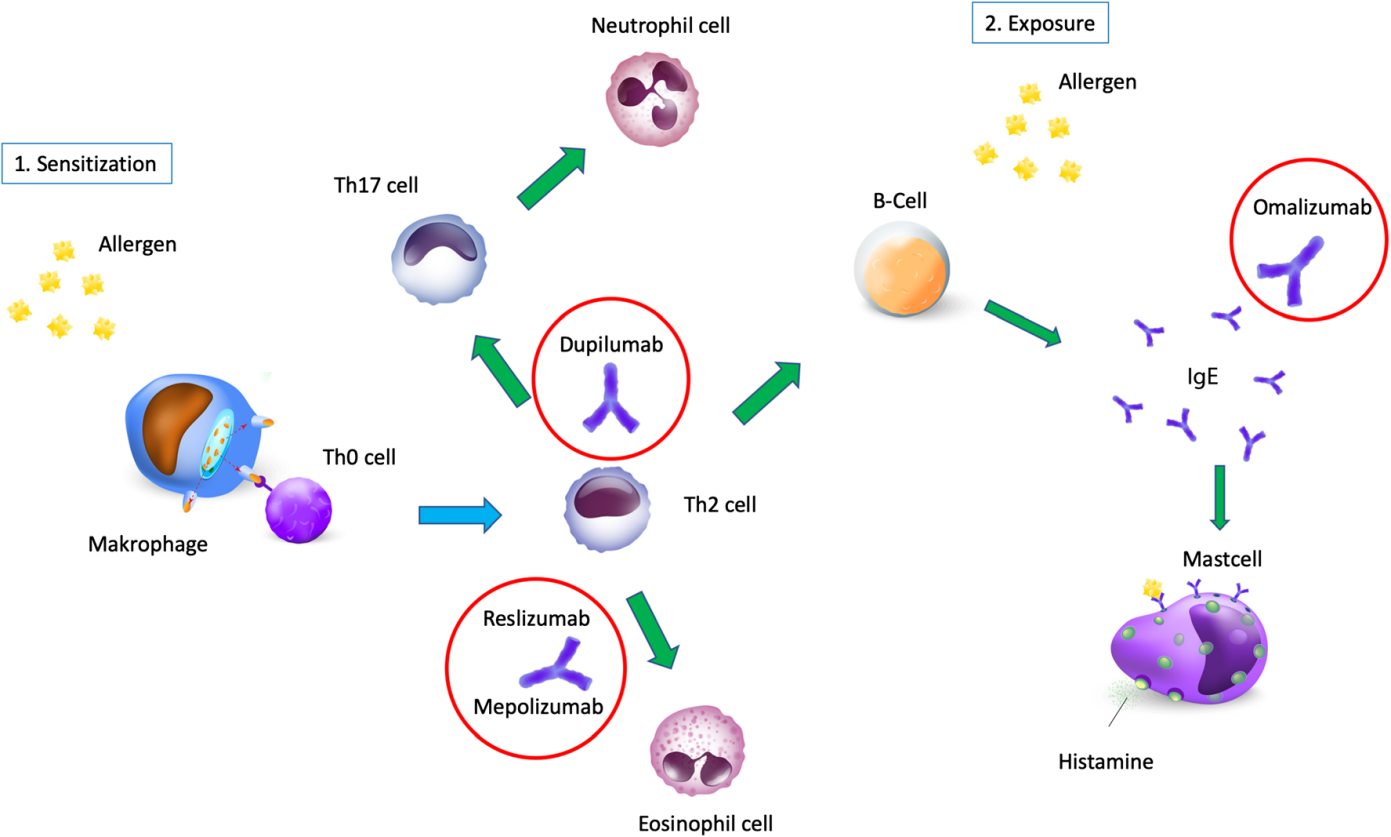
Mechanisms of allergy and the targets of current monoclonal antibodies. A current immunological model of allergy mechanisms and the position at which available biolocials for the treatment of asthma may interfere

For the whole field of asthma and allergy genetics, pharmacogenetics is the hot topic of the moment. This is driven by the development and recent market introduction of numerous biologicals (Fig. 3). “Individualized medicine” is necessary to know which (incredibly expensive) drug should be used in which specific patient. While still in early phase, epigenetic modifications, particularly DNA methylation and miRNAs, may have potential assisting in the stratification of patients for treatment and complement or replace in the future biochemical or clinical tests. First epigenetic biomarkers correlating with the successful outcome of immunotherapy have been reported as described in more detail below and with personalized treatment options being rolled out epigenetic modifications might well play a role in monitoring or even predicting the response to tailored therapy.

### Genetics and epigenetics play a role in the response to classical asthma therapy

As predicted [70], individualized medicine in this first phase which has started now will rather restrict access to drugs than tailor new drugs to individual needs. Using genetic and nongenetic data such as transcriptomics, epigenetics, and metabolomics, asthma and allergy patients may be first characterized as nonresponders to standard therapy as discussed in a previous conceptual paper [71]. In those patients, even though high doses of steroids and other drugs are administered, their disease is not controlled, leading to ever-increasing amounts of drugs with side effects, uncontrolled symptoms, ER visits, and hospital admissions. It is clear that this form of severe allergic diseases needs to be discriminated from “difficult to treat” disease, where patients do not adhere to therapy and the cause for the uncontrolled disease is not a lack of response to treatment but absence of proper management. However, one also has to consider that patients may not adhere to therapy exactly because they see that the prescribed drugs do not work. If patients who are truly unresponsive to standard treatment could be identified easily and characterized early, these patients would be the primary candidates for novel treatments with biologicals.

To achieve this goal, the international consortium on Pharmacogenomics in Childhood Asthma (PiCA) was formed recently. Bringing together studies with GWAS data already available across different countries and ethnicities, this consortium was able to determine a handful of genetic risk factors for an insufficient response to inhaled corticosteroids (ICS) and exacerbations in children and young adults of Caucasian, Hispanic, and African American origin [72]. Especially, a locus on chromosome 22 harboring *APOBEC3B* and *APOBEC3C* was identified in admixed populations and was replicated in follow-up studies of European origin. The gene locus has not been implicated in asthma or allergy in previous studies, but is a biologically plausible candidate as it is involved in innate immunity, virus defense and RNA editing. In the same study, also three other gene loci, which previously had been associated with ICS response in adults, were confirmed.

Epigenetic data investigating drug response is still very scarce, often limited to very small cohorts and even more restricted in the pediatric setting. Inhaled or oral corticosteroid use has been shown to affect epigenetic patterns. Glucocorticoid treatment leads to global loss of histone acetylation through activation of several HDACs and displacement of NF-κB from glucocorticoid receptor (GR) binding sites [73, 74]. Decreased sensitivity to synthetic glucocorticoids has been linked to decreased levels of HDAC2, which deacetylates the glucocorticoid receptor, and might be worsened by passive smoking [75, 76]. Increasing HDAC levels in therapeutic interventions might thus constitute a new way to maximize treatment efficacy, which is also supported by a number of recent findings relevant for the physiopathology of allergic diseases described below.

Systemic exposure to corticosteroids has been found to be associated with differential DNA methylation in whole blood from patients with COPD [77]. There is preliminary evidence that DNA methylation changes might contribute to treatment response as methylation changes in genes including the *OTX2* and the *VVN1* promoter were observed in good but not in poor responders in nasal epithelial cells during treatment [78, 79]. An EWAS using 8-year old children diagnosed with asthma from the BAMSE cohort identified 20 CpGs reaching statistical significance to be associated with any or continuous corticosteroid exposure, but replicating in the STOPPA cohort as well the BAMSE cohort at 16 years of age replicated none of these CpGs [80]. However, more recently, an EWAS performing a meta-analysis of the corticosteroid use in the CAMP, BAMSE, and GACRS cohorts identified two differentially methylated CpGs in the upstream regions of *IL12B* and *CORT* to be associated with absence of severe exacerbations on ICS treatment or absence of oral corticosteroid use, respectively, as a proxy for inhaled corticosteroid response [81]. Of note while not reaching significance in all three cohorts, hypomethylation of *OTX2* was confirmed in the GACRS cohort. However, in contrast to gene expression signatures, no DNA methylation-based biomarker has so far been identified to be predictive for response to corticosteroids [82, 83]. These DNA methylation markers might nonetheless assist in molecularly defining patients unresponsive to corticosteroids having difficulties controlling their asthma. A recent EWAS also investigated the effect of sympathomimetic bronchodilators (albuterol) on the DNA methylation patterns in nasal epithelial cells and identified 130 CpGs associated with the treatment [30]. However, further validation of these findings is required.

Very few studies have analyzed miRNAs in relation to given treatments in allergic diseases. Treatment with inhaled corticosteroids (ICS) had modest effects on miRNA expression patterns and changed the expression of nine miRNAs in a small cohort of steroid-naïve asthma patients [84]. miR-21 is a well-known miRNA in allergic diseases and induces polarization of naïve T cells towards the Th2 lineage and the synthesis of the associated pro-inflammatory cytokines. Higher levels of miR-21 were found in children resistant to inhaled corticosteroids, compared to children sensitive to ICS [85]. However, as miR-21 levels in ICS-resistant children were similar to patients without ICS, the decreased levels in ICS sensitive patients are probably a result of the improvement of their asthmatic status rather than predisposing therapy to success. Similarly, miRNA changes correlated with the use of oral steroids or antileukotriene therapy [86] and allergen induced changes in miRNA expression were reverted by glucocorticoids in patients with eosinophilic esophagitis [87].

Using admixed populations (SAGE II and GALA II populations) for screening, the American TopMED consortium recently identified genetic determinants for bronchodilator response (BDR) associated with lung capacity (*DNAH5*), immunity (*NFKB1* and *PLCB1*), and beta-adrenergic signaling (*ADAMTS3* and *COX18*) [88]. Thus, these signals may be of value across different ethnicities and replication could be expected in populations from different parts of the world. BDR also changes over time, a fact that is well known to pediatricians who often observe a poor response to ADRB2 agonists in babies and very young children. Interestingly, different genetics factors may be responsible for bronchodilator response in younger versus older children and adults. That is suggested by data of the CAMP study when SNPs near *SPATS2L* and *ASB3* demonstrated strongest associations with BRD in early childhood throughout adolescence, and a large decrease in effect size afterwards [89].

Also recently, the genetic basis of moderate to severe asthma was investigated in a massive study involving more than 10,000 patients and almost 50,000 controls [89]. This can be viewed as a pharmacogenetic study, as cases were patients not adequately controlled with low or medium levels of ICS. In addition to known variants already detected in study addressing general asthma previously, three novel loci harboring *MUC5AC*, *GATA3*, and *KIAA1109* with convincing biological plausibility emerged. Altered expression of the pathogenic mucin MUC5AC potentially contributes to mucus plugging and airway obstruction, GATA3 is a transcription factor linked to the T cell response in asthma and eosinophilia, and the *KIAA1109* locus has previously been associated with allergic sensitization. Presence of risk alleles in these genes may thus help to identify nonresponders to conventional therapy and candidates for advanced therapies.

In general, the combination of ICS and LABA is the cornerstone of therapy in moderate to severe asthmatics and a recent study suggest that the response to combination therapy is under genetic control [90]. Interestingly, many glucocorticoid-induced genes were shown to be independently induced by LABA. Variance in target transcription could be explained by gene-specific control by glucocorticoid receptor- and LABA-activated transcription factors, as the authors suggested. Thus, failure to improve to combination therapy in asthma may be a polygenic trait such as asthma itself. Further support for this theory comes from a recent analysis of mutations in the G-coupled receptor family, which SABA, LABA, and other asthma therapies target. It showed a great genetic variability in this pharmacologically important group of receptors, which may explain the interindividual variability in drug response [91].

### Epigenetic changes during allergen immunotherapy

Allergen immunotherapy aims at inducing tolerance to a given allergen or at least sustained unresponsiveness. Depending on the route of application different protocols have been developed including subcutaneous (SCIT), sublingual (SLIT), oral (OIT), and more recently epicutaneous (EPIT) immunotherapy. Allergic sensitization has been shown to alter genes involved in the Th1/Th2 balance in experimental models of asthma [92]. Furthermore, the outcome of immunotherapy can be improved administrating synthetic microRNA mimics of anti-inflammatory miRNAs concurrent with the immunotherapy in a mouse model of allergic rhinitis [93]. In the field of food allergy, induction and maintenance of tolerance to antigens requires the generation of antigen-specific regulatory T-cells (Tregs). Demethylation of the Treg-specific demethylated region (TSDR) of *FOXP3* is a pre-requisite for the stable maintenance of the suppressive properties of Tregs [94, 95]. Demethylation is induced by immunotherapy, and methylation levels remain lower in individuals that show sustained unresponsiveness to allergens such as peanut or milk [96, 97]. Demethylation of *FOXP3* might therefore be a prerequisite for successful immunotherapy. Although the number of individuals analyzed was low in both studies, DNA methylation analysis of the TSDR can be considered as a promising biomarker for monitoring the response to immunotherapy as well as the induction of potential tolerance. Similarly, we have recently shown in a mouse model of epicutaneous immunotherapy for peanut allergy that *Foxp3* methylation was reduced upon successful EPIT, while methylation of the Th2 key transcription factor *Gata3* was specifically increased in splenic CD4^+^ IL4^+^ T cells [98]. In contrast, OIT induced only demethylation of *Foxp3*, but not methylation of *Gata3*, suggesting that the latter might be important to maintain the level of sustained unresponsiveness and protection against sensitization to a second allergen observed in EPIT. In addition, OIT to peanut allergy has shown to induce the differentiation of novel CD4^+^ T cell subsets [99]. Although these have so far only been characterized at the transcriptional level, it is highly likely that these novel CD4^+^ T cell subsets also contain distinct epigenetic profiles, which could provide further markers correlating with sustained unresponsiveness.

Further evidence for an epigenetic modification of the Th cell polarization comes from a recent study analyzing PBMCs from children with allergic asthma following a three-year dust mite allergen-specific immunotherapy (Der p) [100]. Allergic sensitization has been shown to alter genes involved in the Th1/Th2 balance to yield a pro-Th2 phenotype in experimental models, which was also observed in asthmatic patients following Der p challenge [92, 101]. Patients treated with Der p immunotherapy showed increased DNA methylation at the *IL4* promoter suggesting inhibition of the Th2 pathway in children undergoing immunotherapy compared to allergic asthmatics without immunotherapy [100].

### Genetics and epigenetics and the therapy with biologicals

Also, the response to biologicals shows a great variability. The better patients are characterized by molecular biology, the better their response is to biologicals in preventing asthma exacerbations, as summarized very elegantly in a recent review [102]. However, this characterization in current studies is rather primitive: Those patients with elevated eosinophils and/or elevated FeNO respond better to all kinds of biologicals, suggesting that this is a just a crude measure of true severity and not a molecular characterization of the disease. This is what is missing so far and a first step would be to understand why some patients are not responding to ICS therapy. Exactly that is the goal of ongoing EU funded studies such as the SysPharmPediA and PERMEABLE consortia, which aim to identify biomarkers in severe asthmatics that define corticoid resistance and in a next step, to identify specific susceptibility for specific biologicals. Genetics may be of help in that quest as genetic determinant may contribute to the specific responsiveness to certain biologicals.

For anti-IL-5, a GWAS using data from clinical studies on mepolizumab (DREAM and MENSA), was recently published, and there is a clear trend (which is just not significant after correction for multiple testing) towards an association between the prevention of exacerbation on mepolizumab and a locus on chromosome 6 harboring *UTRN* and *EPM2A* and a further locus on chromosome 9 which included different type 1 Interferon genes such as *IFNA14* [103]. Why the authors, who are mainly current or former employees of a pharmaceutical company, do not follow up on these highly suggestive and plausible associations remains unclear. Especially, as these data has a potential to stratify patients for their responsiveness to anti-IL5 therapy and could be used to spare unresponsive patients from an unnecessary use of these antibodies while others could be identified as having a rather good chance to respond to this biological. Interestingly, such studies, where biomarkers or predictors for the response to omalizumab, the monoclonal antibody against IgE, which is on the market since 2003, would have been investigated, have not been published so far. For dupilumab, the newest monoconal antibody directed against the receptor chain shared by IL-4 and IL-13, such studies for genetic susceptibility also do not exist. However, we showed in our previous work [104], that the concomitant presence of multiple polymorphisms in the IL-4/IL-13 pathway in an individual may contribute significantly to the development of a mainly allergic form of asthma. Carrying SNPs associated with asthma risk in three different genes of the pathway increased the risk up to 30-fold in our study population of German children, affecting approximately 2–4% of the population under investigation. Thus, based on molecular mechanisms already identified, one could speculate that exactly these asthma patients carrying such multiple SNPs in the pathway are those that may respond to dupilumab treatment.

Epigenetic data supporting the use of biologicals is still rare. At least two EWAS have found differential methylation of CpGs in the IL5 receptor [27, 34], which is targeted by the monoclonal antibody benralizumab. A CpG in this gene has also been found to be associated with allergic sensitization [105]. It would therefore be interesting to investigate the associations or correlation between the degree of methylation of this gene and the response to benraluzimab.

As described above, eosinophils from asthmatic children showed an altered DNA methylation profile suggesting a differential activation state in the recent multi-cohort study from the MEDALL consortium [26]. These findings provide an interesting basis to investigate how eosinophil targeting/depleting therapies with anti-IgE-, anti-IL-13-, or anti-IL-5Ra-based antibodies will modify the DNA methylation landscape in eosinophils and if there is any correlation between the response to therapy and the pre-treatment epigenetic profile. Epigenetic profiling could also yield in the future biomarkers, which could assist in the classification and selection of patients that would profit most from a potential biological treatment or on the other hand for which little improvement could be expected.

### A potential next step: targeting epigenetic enzymes in allergic diseases

In addition to the above described implication of histone deacetylases in the response to corticosteroids, a number of recent studies have demonstrated a beneficial effect of blocking histone deacetylases in allergic diseases. The blocking of HDAC activity (using a pan HDAC inhibitor JNJ-26481585) restored the integrity of the nasal epithelium from patients with allergic rhinitis and restored mucosal function and prevented the development of airway inflammation and hyperresponsiveness in experimental models [106] Furthermore, the HDCAi trichostatin A improved atopic dermatitis in a mouse model reducing notably expression for Th2, but not Th1 cytokines [107]. Although constituting a novel and promising approach, the use of HDAC inhibitors (HDACi) has yielded conflicting result with some studies pointing to enhanced inflammation thus requiring further investigation of the use of this treatment [108–110]. However, as HDACs and HATs (de)acetylate a large number of targets and are involved in a multitude of cellular pathways, inhibition or modulation of these processes might provoke undesired adverse effects requiring the development of more selective HDAC inhibitors targeted to specific cell populations. The Polycomb protein Ezh2, the main H3K27me3 methylase, has been shown to be critically involved in the differentiation and plasticity of CD4^+^ Th1 and Th2 cells controlling the correct expression of the key transcription factors *Tbx21* and *Gata3*, promoting Th1 responses and the loss resulted in the accumulation of memory Th2 cells [111]. Ezh2 further prevents the development of pathological NKT cells preventing a spontaneous asthma-like phenotype in experimental models [112]. First results show also the possibility of improving allergic inflammation and airway hyperresponsiveness in experimental asthma models by administrating a H3K27Me3 specific histone demethylase inhibitor (GSK-J4 [113]).

## Conclusions

Taken together, there are a still a number of open questions in asthma to which genetics and epigenetics may give answers to and thus, at the end, may even help the patients. Despite the rapid progress in recent years, there are still numerous challenges for the interpretation of existing and future data. In epigenetics, it is not yet clear which tissue and cell types are best suited for analysis [114]. Most analyses have been performed in blood immune cells, but respiratory epithelial cells from the nose or bronchi have also been studied, show much better discrimination between asthmatics and controls. For genetics and epigenetics, the often imprecise definition of the underlying clinical phenotype (e.g., how and by whom asthma or other allergies were exactly diagnosed) also makes interpretation difficult and makes previous study results only partially comparable. Furthermore, longitudinal studies with samples available prior to the onset of symptoms, e.g., birth cohorts with repeated biological samplings are required to better investigate causality and relationships between markers, onset, and course of disease.

Technical progress led and leads to ever more voluminous, high-dimensional multi-omic data sets. The future challenge will be to analyze and integrate these data sets in order to obtain a systems medicine view of the molecular processes underlying the development and progression of allergic diseases [115]. First big data and multi-omic studies have shown that allergic diseases are very complex and dynamic and that further systems biology studies are required. Advances in machine learning algorithms and artificial intelligence are timidly making their first steps in the field of allergy and refine epigenetic signatures [116], but their power remains limited due to the absence of sufficiently large data volumes.

With the choice of biologicals now available for treatment, the prediction of treatment response and the matching of patients to specific therapies becomes crucial for the patient as well as for the health system. Thus, a better understanding of allergy and asthma mechanisms in the individual patient and to have biomarkers for decision making are now needed more than ever. Epigenetics and genetics have the potential to make substantial contribution and the analysis of epigenetic changes will have an important role in designing a customized (immune) therapy, preventing side effects and defining an optimal therapy duration. This will ultimately contribute to improving the quality of life of allergy patients.
